# Diabetes-Induced Organ Damage: Cellular Mechanisms and Therapeutic Opportunities

**DOI:** 10.3390/cells15191723

**Published:** 2026-09-22

**Authors:** Mohammed S. Razzaque

**Affiliations:** 1 Department of Medical Education, School of Medicine, University of Texas Rio Grande Valley (UTRGV), Edinburg, TX 78541, USA; mohammed.razzaque@utrgv.edu or msr.nagasaki@gmail.com; 2 Faculty of Medicine, Universiti Kuala Lumpur Royal College of Medicine Perak (UniKL RCMP), No. 3, Jalan Greentown, Ipoh 30450, Perak, Malaysia

## 1. Cellular Events and Complications of Diabetes

Diabetes mellitus is a metabolic disorder characterized by insulin resistance and impaired pancreatic beta-cell function, resulting in sustained hyperglycemia. In type 1 diabetes, autoimmune destruction of beta cells mediated by autoreactive T cells, inflammatory cytokines, e.g., interleukin-1 beta (IL-1β), interferon gamma (IFN-γ), and tumor necrosis factor alpha (TNF-α), and oxidative stress lead to absolute insulin deficiency. The pathogenesis of type 2 diabetes involves insulin resistance in peripheral tissues and progressive pancreatic beta-cell failure mediated by glucotoxicity, lipotoxicity, ER stress, and amyloid accumulation. Persistent hyperglycemia drives the progression of diabetic complications by activating multiple pathogenic pathways. These include the polyol pathway, advanced glycation end-product (AGE) formation and receptor for advanced glycation end products (RAGE) activation, protein kinase C (PKC) signaling, and increased hexosamine pathway flux, which collectively promote oxidative stress, inflammation, vascular dysfunction, and dysregulated gene expression. These mechanisms culminate in mitochondrial dysfunction, reactive oxygen species (ROS) overproduction, NF-κB-mediated inflammation, and endothelial dysfunction, ultimately leading to various debilitating irreversible complications.

A prolonged glucose burden causes widespread structural damage to blood vessels, nerves, and vital organs, leading to systemic organ dysfunction. Diabetes-related damage is commonly associated with microvascular complications, including retinopathy, nephropathy and neuropathy, and macrovascular complications, such as atherosclerosis, cardiovascular disease, and cerebrovascular disorders. As mentioned, chronic inflammation, oxidative stress, endothelial dysfunction, metabolic dysregulation, and AGE accumulation are major underlying causes of these complications [1]. While the incidence of diabetes continues to increase globally, understanding the cellular events and molecular mechanisms promoting diabetic complications has become increasingly important for developing diagnostic and therapeutic strategies aimed at reducing long-term complications and improving disease outcomes.

## 2. Challenges in Diabetic Treatment

The management of diabetes mellitus remains challenging because diabetes is a complex and multifactorial disease that affects multiple organs and metabolic pathways. Although current therapies such as insulin, oral hypoglycemic agents, sodium-glucose cotransporter 2 (SGLT2) inhibitors and glucagon-like peptide-1 (GLP-1) receptor agonists can improve glycemic control, a significant number of patients develop long-term complications despite well-controlled glucose levels. Major challenges include progressive β-cell dysfunction, insulin resistance, poor treatment compliance, risk of hypoglycemia, drug-associated adverse effects, and individual variability in therapeutic responses. Disease management is further complicated by the need for repeated injections, high treatment costs, limited healthcare access, and suboptimal patient adherence. Emerging research also highlights that glucose control alone may not fully address the inflammatory, metabolic, and vascular abnormalities related to diabetic complications, emphasizing the need for more personalized, multitarget, and organ-protective therapeutic strategies. Current therapeutic options for diabetes include medications, emerging stem cell therapy, and approaches that target the maintenance of cellular homeostasis. Despite these advances, important gaps remain in understanding the subcellular mechanisms of diabetes-induced organ damage and in the development of effective targeted therapies. The contributions in this Special Issue of *Cells*, entitled “Diabetes-Induced Organ Damage: Cellular Mechanisms and Therapeutic Opportunities,” provide important insights into the underlying diabetes-induced complications and highlight emerging therapeutic strategies aimed at modulating organ damage associated with prolonged diabetes.

## 3. Key Contribution to This Special Issue

This Special Issue includes seven research articles and five in-depth review articles that discuss diabetes-induced organ damage and emerging therapeutic strategies to reduce the disease burden of diabetes, including the role of micronutrients in delaying the onset of diabetic complications. Moon et al. reported that norrin reduces retinal ganglion apoptosis in diabetic retinopathy by restoring the balance of two key retinal factors, vascular endothelial growth factor (VEGF) and pigment epithelium-derived factor (PEDF), which are dysregulated in diabetic retinopathy [2]. Investigators using diabetic mouse models have shown that norrin attenuates hyperglycemia-induced neurodegenerative processes that lead to retinal ganglion apoptosis and that norrin reduces hyperglycemia-induced microvascular leakage and retinal ganglion apoptosis by restoring the dysregulated expression of VEGF and PEDF [2]. This study supports the idea that diabetic retinopathy is not only a vascular disease but also involves important neurodegenerative changes in the retina [3]. This work adds to prior evidence that norrin has neuroprotective and vascular-stabilizing effects on retinal injury [4]. Norrin has the potential to become a candidate therapeutic target for diabetic retinopathy, especially if future studies confirm its safety and efficacy in larger preclinical and clinical settings [5].

Microvascular complications, including diabetic retinopathy, nephropathy, and neuropathy, are major contributors to chronic disease burden (Figure 1), and developing targeted therapies to prevent or delay the progression of diabetic microvascular complications remains a significant clinical challenge. In an article by Abu El-Asrar et al., ADAMTS13 was identified as a potentially important therapeutic regulator of diabetic retinopathy, especially the proliferative form of diabetic retinopathy. A study demonstrated that endogenous ADAMTS13 expression is reduced in diabetic retinas, whereas treatment with recombinant ADAMTS13 significantly improved retinal vascular integrity and function in diabetic rat models [6]. ADAMTS13 reduced VEGF-induced endothelial migration and in vitro angiogenesis. This article supports the idea that diabetic retinopathy is not only a VEGF-driven angiogenic disease but also a thrombo-inflammatory disorder that includes active endothelial damage, leading to platelet—endothelial interactions, in proliferative diabetic retinopathy patients. The results are promising, but the long-term safety of intravitreal ADAMTS13 is unknown, and excessive anticoagulant activity could theoretically increase the risk of bleeding. Despite these potential limitations, this study connects vascular injury, inflammation, thrombosis, and endothelial dysfunction into one mechanistic pathway centered on the interaction between ADAMTS13 and von Willebrand factor, which is an interesting observation that deserves further clinical exploration.

In a complementary review article, Seo et al. explained why diabetic retinopathy is a multifactorial neurovascular inflammatory disease rather than simply a late-stage vascular complication of diabetes [7]. This review emphasizes that persistent hyperglycemia initiates several interconnected pathological events, including oxidative stress, chronic inflammation, endothelial dysfunction, neurodegeneration, microvascular injury and breakdown of the blood–retinal barrier. Eventually, the retina becomes progressively damaged through both vascular and neural dysregulation. While current anti-VEGF therapies remain clinically useful, they do not completely prevent disease progression. Future therapies may simultaneously target inflammation, oxidative stress, vascular dysfunction, neurodegeneration, and angiogenesis; this highlights the need for a broader therapeutic strategy focused on system-level retinal protection rather than isolated pathway suppression.

In a separate research article, a novel small molecule, ST32da, derived from Salvia miltiorrhiza, was identified as a potential therapeutic for diabetic nephropathy associated with obesity and type 2 diabetes [8]. The central idea of this study is that stimulating activating transcription factor 3 (ATF3) can suppress inflammatory and metabolic stress pathways that drive kidney injury in diabetes. ST32da increased ATF3 expression and substantially improved renal pathology in mouse models of multiple obesity-related diabetic nephropathy; the beneficial effects included reduced albuminuria, kidney inflammation, and fibrosis, along with improved glomerular architecture, decreased podocyte injury, and improved renal histology. ST32da treatment also reduced the expression of inflammatory and profibrogenic factors, including interleukin-6 (IL-6), TNF-α, and transforming growth factor-beta (TGF-β). One of the most important findings is that ATF3 expression is reduced in the kidneys of patients with diabetic nephropathy and that the loss of ATF3 exacerbates disease severity in mouse models. These findings suggest that patients with low ATF3 activity and strong inflammatory signatures may particularly benefit from ATF3-targeted therapies. This study identifies ATF3 as a potentially protective regulator in obesity-related diabetic nephropathy and introduces ST32da as a promising anti-inflammatory, antifibrotic, and metabolically protective therapeutic candidate.

Another reported study investigated how Imeglimin affects diabetic atherosclerosis, particularly the formation of macrophage foam cells, which is an essential process in plaque development. Using both cultured macrophages and diabetic apolipoprotein E (ApoE)-deficient mice, the investigators provided evidence that Imeglimin may have direct cardiovascular-protective effects beyond lowering glucose levels [9]. Importantly, Imeglimin significantly inhibited the conversion of macrophages into foam cells under diabetic conditions. Mechanistically, Imeglimin increased the expression of ATP-binding cassette transporter A1 (ABCA1) and ATP-binding cassette transporter G1 (ABCG1), which are major cholesterol efflux transporters that remove cholesterol from macrophages and transfer it to HDL particles [10]. Imeglimin increased AMP-activated protein kinase (AMPK) phosphorylation, increased ABCG1 expression through AMPK, and suppressed CD36 expression through AMPK signaling. Notably, AMPK is considered a master regulator of cellular energy metabolism, lipid metabolism, inflammation, and mitochondrial function [11]. This links Imeglimin to broader metabolic and anti-inflammatory pathways already implicated in cardiovascular protection [12]. Furthermore, in ApoE-deficient mice with streptozotocin-induced diabetes, Imeglimin significantly reduced the aortic plaque area, lipid deposition, and fasting glucose and low-density lipoprotein (LDL) cholesterol levels. The mice also showed increased ABCG1 expression in liver tissue and aortic tissue, demonstrating that the in vitro cellular responses translated into reduced vascular function in vivo. A major conceptual contribution of this study is the idea that Imeglimin may directly protect blood vessel and macrophage functions independent of its glucose-lowering effects. These findings imply that Imeglimin could eventually join a growing class of antidiabetic agents with cardiovascular-protective properties. This parallels the evolution of SGLT2 inhibitors and GLP-1 receptor agonists, which were initially developed for use as glucose-lowering drugs but were later shown to reduce cardiovascular risk. Notably, the mouse atherosclerotic process substantially differs from human plaque-forming biology [13].

A related study demonstrated how chronic high-glucose exposure alters the bone extracellular matrix (ECM), especially the collagen network, using an ex vivo embryonic chicken femur model [14]. Increased synthesis and accumulation of ECM is a common feature of long-standing diabetic complications, as documented in both human and experimental studies [15,16,17]. This research is important because it shifts attention from bone mineral density alone toward the quality and organization of bone collagen in diabetes mellitus-associated skeletal fragility. High glucose profoundly disrupts bone collagen organization through abnormal collagen fibril alignment, altered fibril diameter, disrupted spatial organization, impaired fibrillar maturation, and densely packed but poorly organized collagen architecture. Using ATR-FTIR spectroscopy, the results of this study revealed that high glucose levels impaired collagen crosslinking and matrix integrity through increased nonenzymatic glycation, impaired collagen crosslinking, reduced collagen integrity, and altered ECM composition. Since collagen crosslinking determines bone elasticity, resistance to microdamage, and mechanical toughness, these results support the idea that excess glucose causes pathological glycation and the accumulation of AGEs that stiffen and weaken bone tissue. Notably, translating findings from an ex vivo embryonic chicken model to human adult bone biology is challenging; therefore, the precise clinical relevance and translational potential remain uncertain.

An accompanying review article explains how type 1 diabetes disrupts the integrated relationship between muscle and bone in children and adolescents [18]. Type 1 diabetes disrupts normal muscle–bone crosstalk in children and adolescents by reducing bone mineral accumulation, impairing peak bone mass development, altering muscle composition, reducing muscle strength, and increasing fracture risk. A major conceptual point is that fracture risk in type 1 diabetes cannot be explained solely by reduced bone mineral density, as abnormalities in bone microarchitecture, collagen crosslinking, cortical geometry, trabecular structure, and bone turnover could collectively contribute to the pathology. This review highlights the mechanical dependence of bone on muscle loading and highlights that muscle dysfunction contributes directly to skeletal abnormalities. Physical activity is protective but underutilized, and exercise is among the strongest nonpharmacologic interventions for preserving bone health, muscle mass, insulin sensitivity, and mitochondrial function [19,20]. The broader implication is that future pediatric type 1 diabetes care may focus on preserving musculoskeletal development, bone quality, muscle function, and lifelong skeletal resilience rather than concentrating exclusively on controlling glucose levels alone.

Neuropathy is among the common complications of type 1 diabetes, and prior studies have shown its association with demyelination, including alterations in myelin basic protein (MBP). Epidemiological evidence also indicates a bidirectional association, with individuals with type 1 diabetes at increased risk of multiple sclerosis, and vice versa. In an accompanying article, sequence analyses revealed similarities in immune-related receptors and molecular mimicry between type 1 diabetes and multiple sclerosis; BLAST (National Library of Medicine, Bethesda, MD, USA) identified highly similar T-cell receptor (TCR) sequences across both conditions, whereas LALIGN showed homology between MBP and type 1 diabetes-associated antigens. Similarly, antibodies against insulin, PTPRN (IA-2), and GAD1 cross-reacted with MBP, and most TCRs and serum samples from patients with type 1 diabetes recognized MBP, unlike those from healthy controls. This study revealed that cross-reactivity between type 1 diabetes-related antibodies and TCR with MBP may help explain the pathogenesis of type 1 diabetes neuropathies and the increased co-occurrence of these two diseases [21].

A review article on diabetic neuropathy discusses the progressive, length-dependent degeneration of peripheral axons caused by interactions among metabolic, inflammatory, and vascular disturbances [22]. Diabetic neuropathy disrupts cytoskeletal integrity and axonal transport in long peripheral axons, with AGE–RAGE signaling playing a central role in driving distal “dying-back” degeneration. Therapeutically, these mechanisms highlight the need to move beyond symptomatic management toward strategies that preserve axonal integrity, maintain transport systems and limit AGE–RAGE signaling. Accordingly, future research should integrate molecular pathway analysis with structural and functional nerve assessments, such as nerve fiber density and conduction studies, to better link mechanistic insights with clinically meaningful outcomes [22]. Another informative review article provides an overview of the underlying pathophysiological mechanisms of diabetes and discusses potential therapeutic strategies [23]. This explains the cellular and molecular events that drive insulin resistance and beta-cell dysfunction. This review also details current and emerging therapeutic approaches, ranging from conventional antidiabetic agents to novel targets aimed at addressing the root causes of disease pathogenesis to increase insulin sensitivity.

A noteworthy study provides evidence to support the growing concept of “diabetic lung disease” as a real diabetic complication, not merely a secondary consequence of obesity [24]. Diabetic mice develop pulmonary inflammation, structural damage to the alveoli, epithelial injury, and pulmonary fibrosis. Researchers have shown that swimming, resistance exercise, and high-intensity interval training significantly improve pulmonary pathology by reducing inflammatory cytokines, oxidative stress, macrophage infiltration, fibrosis markers, and collagen deposition [24]. One of the important mechanistic findings of this study is that exercise shifted lung macrophages from a pro-inflammatory M1 phenotype toward an anti-inflammatory M2 phenotype, suggesting that exercise may “reprogram” immune responses in diabetic tissues, including in the lung (Figure 2). Notably, swimming had the strongest anti-inflammatory effects, whereas high-intensity interval training had the strongest antifibrotic signaling effects. Exercise may become a targeted therapy for diabetic lung disease and may eventually be viewed as a mechanistic therapy rather than merely a form of lifestyle advice. However, the study was conducted on mice, and human lung physiology may differ substantially; thus, the translation of exercise intensity to humans is uncertain. At this point, the broader implication is that exercise may eventually become a precise, organ-protective therapy for diabetic complications beyond glucose control alone [25,26].

In addition to lifestyle modification and exercise, the importance of nutrition is also highlighted in this Special Issue. Zinc is actively involved in glucose metabolism and insulin signaling, and a review article details the complex relationship between zinc and diabetes mellitus, with potential therapeutic relevance in reducing diabetic complications [27]. In pancreatic β-cells, zinc stabilizes insulin hexamers, supports insulin crystallization and storage, regulates insulin secretion, and influences β-cell survival. Both type 1 and type 2 diabetes are frequently associated with altered zinc metabolism. Diabetic patients may exhibit lower serum zinc levels, increased urinary zinc excretion, impaired zinc absorption, and intracellular dysregulation of zinc homeostasis. Hyperglycemia-induced polyuria partly contributes to zinc loss. Imbalances in zinc may contribute to metabolic dysfunction and diabetic complications, whereas zinc supplementation has been associated with improved fasting glucose and HbA1c levels, enhanced insulin sensitivity, reduced oxidative stress markers, improved lipid profiles, and a lower risk of diabetic complications (Figure 3). This review reinforces the idea that metabolic disease management may increasingly combine pharmaceuticals, nutritional optimization, anti-inflammatory strategies and lifestyle modifications. Zinc supplementation could eventually become an adjunctive strategy in selected patients.

## 4. Conclusions

In summary, this Special Issue integrates basic and translational research to provide a more unified understanding of diabetes-induced cellular and tissue damage across multiple organ systems. By elucidating shared molecular and cellular mechanisms, these studies are likely to assist the development of innovative targeted therapies to delay the progression of metabolic complications to reduce the disease burden of diabetes. The articles in this Special Issue provide a compelling overview of the complexity of diabetes progression and its complications, along with emerging therapeutic potential, including ADAMTS13, Imeglimin and ST32da. Collectively, the results underscore the value of investigating model systems in advancing mechanistic insight and guiding future interventions to reduce the disease burden of diabetes.

## Figures and Tables

**Figure 1:**
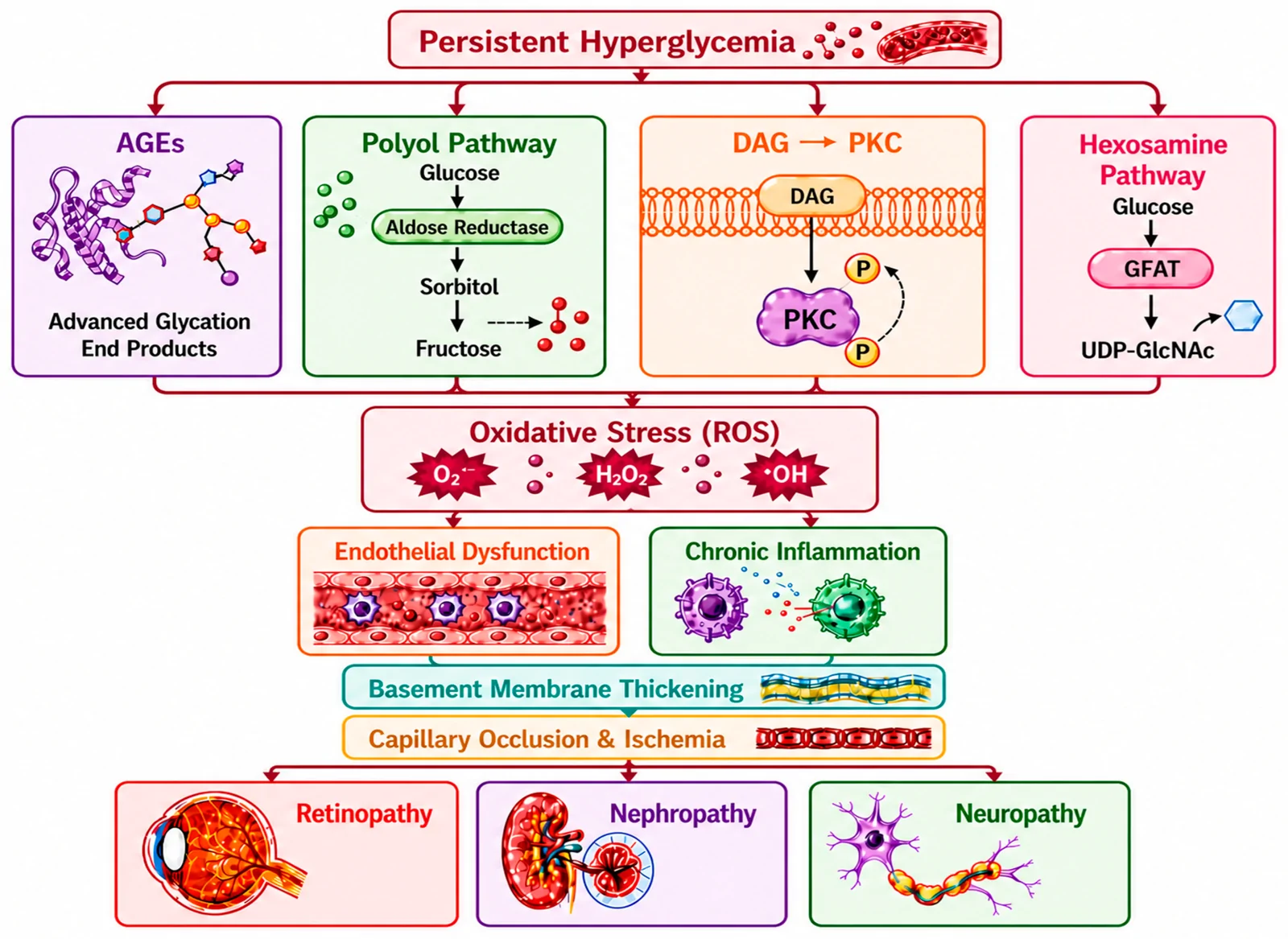
Persistent hyperglycemia initiates a network of interconnected metabolic and molecular pathways, including advanced glycation end product (AGE) formation, increased polyol pathway flux, protein kinase C (PKC) activation, hexosamine pathway activation, and oxidative stress. Together, these processes promote chronic inflammation, endothelial dysfunction, and progressive microvascular injury, ultimately leading to diabetic retinopathy, nephropathy, and neuropathy, which account for a substantial portion of the chronic disease burden associated with diabetes.

**Figure 2:**
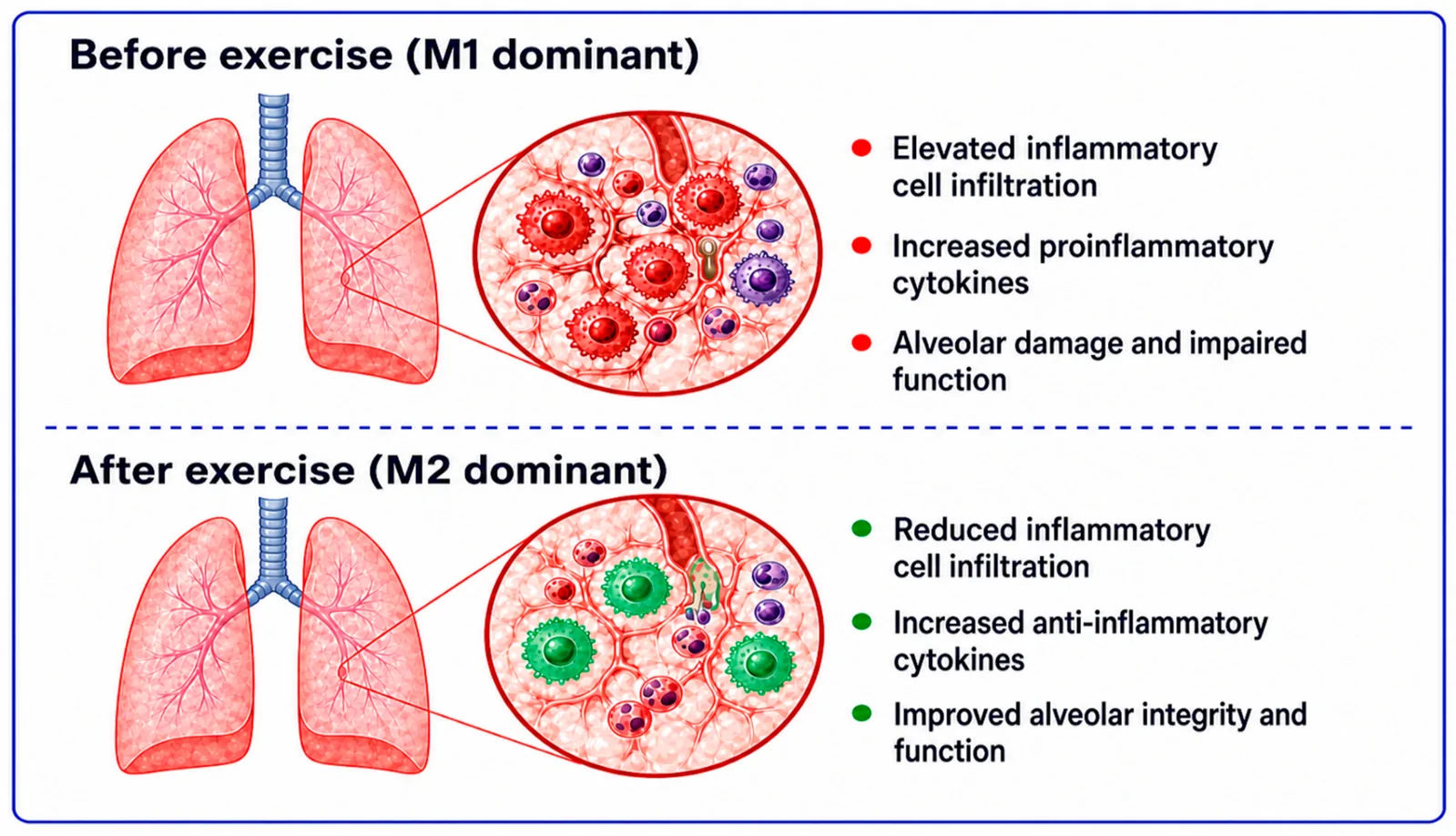
Exercise interventions attenuate diabetic lung inflammation by shifting macrophage polarization from a pro-inflammatory M1-dominant phenotype toward an anti-inflammatory M2 phenotype [24].

**Figure 3:**
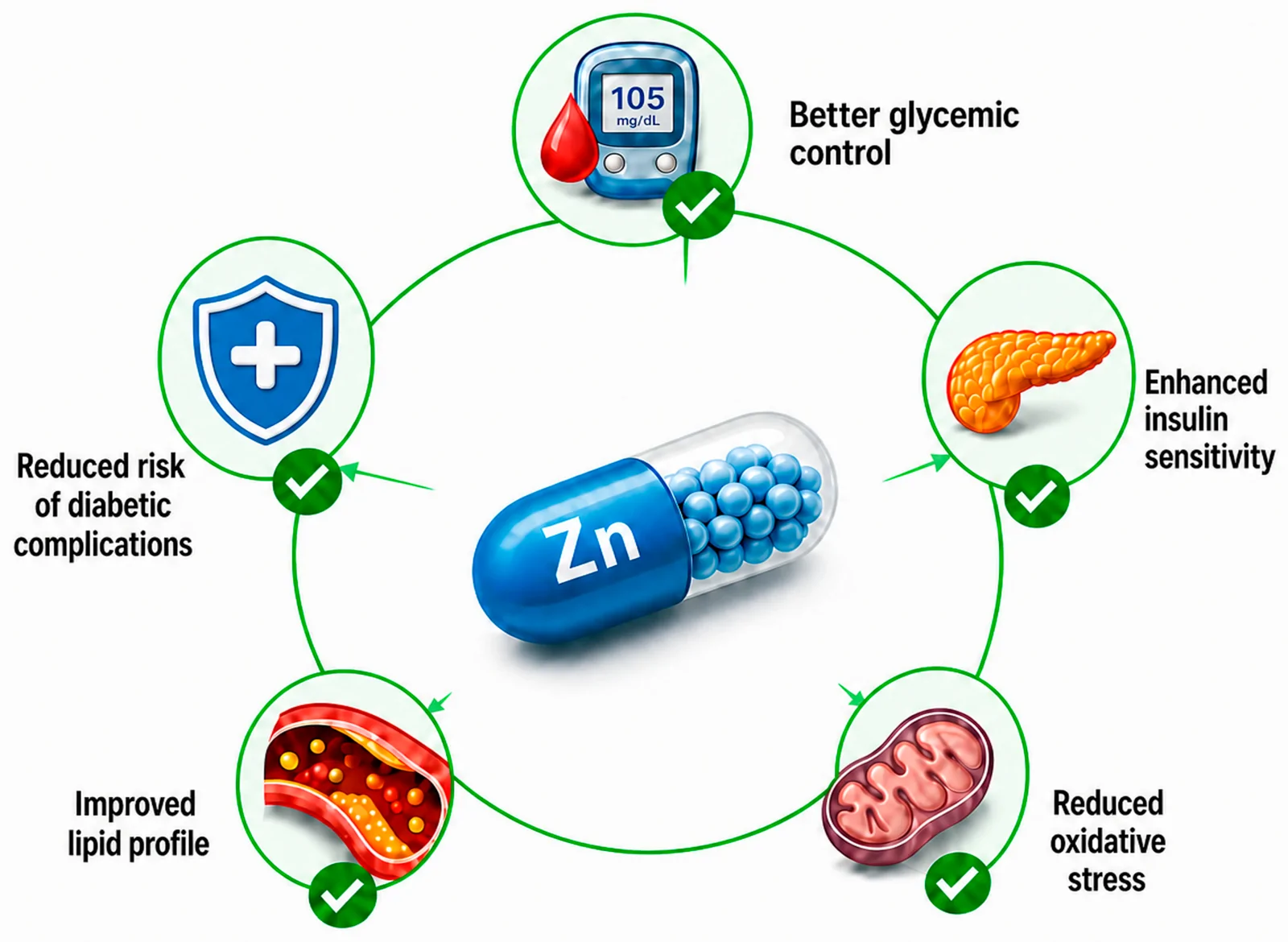
Partial list of documented benefits of zinc supplementation in diabetes [27].

## Data Availability

No new data were created or analyzed in this study. Data sharing is not applicable to this article.
